# Moderate agreement between body mass index and measures of waist circumference in the identification of overweight among 5-year-old children; the ‘Be active, eat right’ study

**DOI:** 10.1186/1471-2431-13-63

**Published:** 2013-04-23

**Authors:** Lydian Veldhuis, Ineke Vogel, Wilma Jansen, Carry M Renders, Remy A HiraSing, Hein Raat

**Affiliations:** 1 Department of Public Health, Erasmus MC – University Medical Center Rotterdam, Rotterdam, the Netherlands; 2 Youth Policy Department, Municipal Public Health Service Rotterdam area, Rotterdam, the Netherlands; 3 Institute of Health Sciences, Faculty of Earth and Life Sciences, VU University Amsterdam, Amsterdam, the Netherlands; 4 Department of Public and Occupational Health, EMGO Institute for Health and Care Research, VU University Medical Center, Amsterdam, the Netherlands

**Keywords:** Body mass index, Waist circumference, Overweight, Preschool child, Pediatrics, Prevalence

## Abstract

**Background:**

Body mass index (BMI) is a common indirect method to assess weight status among children. There is evidence that BMI data alone can underestimate overweight-related health risk and that waist circumference (WC) should also be measured. In this study we investigated the agreement between BMI and WC and BMI and the waist-height ratio (WHtR) when used to identify overweight among children.

**Methods:**

This cross-sectional population-based study uses baseline data from 5-year-olds (n = 7703) collected by healthcare professionals for the ‘Be active, eat right’ study.

**Results:**

According to age-specific and sex-specific cut-off points for BMI (IOTF, 2000) and WC (Fredriks et al., 2005), the prevalence of overweight (obesity included) was 7.0% and 7.1% among boys, and 11.6% and 10.1% among girls, respectively. For the WHtR the 90^th^ percentile was used as the cut-off point. Among boys, observed proportion of agreement between BMI and WC classification was 0.95, Cohen’s kappa 0.58 (95% CI; 0.53-0.63), and proportions of positive and negative agreement were 0.61 and 0.97, respectively. Observed proportion of agreement between BMI and WHtR classification was 0.92, Cohen’s kappa 0.46 (95% CI; 0.41-0.51), and proportions of positive and negative agreement were 0.51 and 0.95. Children identified as overweight according to WC were relatively tall, and children classified as overweight according to the WHtR only were relatively short (comparable results for girls).

**Conclusions:**

There is moderate agreement between BMI and measures of WC on the presence of overweight among 5-year-olds. If BMI data and cut-offs continue to be used, then part of the group of children identified as overweight according to WC and the WHtR will be omitted. Follow-up of the children classified as overweight according to BMI only, WC only, and WHtR only, will give indications whether WC should be measured in addition to BMI or whether WC should only be measured in certain subgroups (e.g. relatively tall or short children) to identify and monitor overweight in children. This may improve early identification and prevention of overweight and overweight-related health problems in children.

## Background

The prevalence of overweight and obesity among children has increased rapidly worldwide [1,2]. The common indirect method of identifying overweight among children is use of the body mass index (BMI) [3-6]. However, it has been indicated that only using BMI data results in an underestimation of health risk, as BMI is an indicator of excess weight relative to height and does not indicate body fat distribution [7]. Waist circumference (WC) is a marker of central fat distribution, and there is considerable evidence that high central fat distribution is associated with an increased risk of metabolic complications, such as insulin resistance, in both adults and children [8-11]. There is, however, at this moment neither international consensus on whether WC should be used in conjunction with BMI to identify overweight among children [1,6,12,13] nor whether WC should be measured as part of a ratio (e.g. waist-height ratio (WHtR)) [11,12,14-17].

In the Netherlands, all children are monitored in a nationwide program at set ages. This is a free service and attendance rate for these well-child visits is 95%. During these regular check-ups, healthcare professionals measure the height and weight of each child [18]. The healthcare professionals also assess whether a child has overweight. They base their decision on the child’s BMI and their clinical judgment by taking into account the child’s stature, ethnicity, and body-fat distribution [19]. However, this clinical judgment is based on the knowledge and experience of the professional and the process can not be standardized and remains arbitrary. A possibility to make the decision less arbitrary is to also measure WC [19]. The question that arises is whether WC should be measured in addition to BMI in monitoring programs to identify all children at increased risk for overweight-related health problems. A first step is to investigate whether BMI and WC agree about children’s weight status. And when BMI and WC disagree about weight status, it is important to have insight in the characteristics of the children not identified as overweight when using only BMI.

We compared BMI versus WC and BMI versus the WHtR in a large population-based sample of 5-year-old children to establish 1) whether BMI and WC agree on weight status of these young children, whether BMI and the WHtR agree on these children’s weight status, and 2) whether there are differences in children’s characteristics (ethnic background, weight, height) between the groups that are overweight according to BMI or WC, and between the groups that are overweight according to BMI or the WHtR. We also examined the clinical judgment of the healthcare professional on the children’s weight status in the groups that are overweight according to BMI, WC or the WHtR.

## Methods

### Design and study population

The present cross-sectional study is embedded in the ‘Be active, eat right’ study, which aims to assess the effects of an overweight prevention protocol as detailed elsewhere [20]. The Medical Ethics Committee of Erasmus MC University Medical Centre Rotterdam approved the study protocol. Nine of the 37 municipal health services in the Netherlands participated in the ‘Be active, eat right’ study. A total of 13,638 parents of 5-year-olds were invited by mail for a well-child visit at one of the nine municipal health services. These parents were also invited to participate in the ‘Be active, eat right’ study and 64.4% provided written informed consent to participate in the study. Baseline data were collected during the 2007–2008 school year, and these data were used for the present study.

Children were excluded from analyses when data were missing on anthropometric measurements (n = 475), on age or sex (n = 98), on weight status according to the clinical judgment of the healthcare professional during the well-child visit (n = 381), on parental educational level (as an indicator of socioeconomic status) or ethnicity (n = 127). After exclusion, a study population of n = 7703 remained.

### Anthropometry, weight status and characteristics

During well-child visits trained healthcare professionals of the municipal health services measured each child’s body weight, height and WC using standardized methods as described in a protocol [19]. Body weight was measured to the nearest 0.1 kg and height to the nearest 0.1 cm. WC was measured over naked skin midway between the lower rib margin and the iliac crest at the end of gentle expiration while the children were in standing position [19]. The healthcare professionals were trained to measure WC using a measuring tape (type of measuring tape; SECA 200).

BMI was calculated by dividing weight (in kilograms) by height (in meters) squared. The weight status of the children according to BMI was assessed using the International Obesity Task Force’s (IOTF) age-specific and sex-specific cut-off points [21]. To assess the weight status of the children according to WC data, we used the age-specific and sex-specific cut-off points for Dutch children as presented by Fredriks et al. [22]. When a child’s BMI or WC value was the same as or higher than the lower-bound cut-off point for overweight for the child’s age and sex, the child was classified as overweight (obesity included).

For the WHtR, no internationally accepted cut-off points are available. We used the 90^th^ percentile within our total study population at baseline (n = 8784) as the lower-bound cut-off point for having overweight (obesity included). The healthcare professionals reported their clinical judgment on the weight status of the children. This judgment was based on the child’s stature, ethnicity and body-fat distribution [19,23,24].

We obtained information about the child’s age, sex, ethnic background, and parental educational level from a questionnaire completed by the parents. A child was considered to be of non-Dutch ethnic background when at least one of the parents was born abroad (according to the definition of Statistics Netherlands [25]). Educational level of the parents was recorded in three categories according to the Dutch standard classification as defined by Statistics Netherlands [26]: high level (academic higher education/university education, higher professional education), mid level (pre-university education, senior general secondary education, and senior secondary vocational education), and low level (preparatory secondary vocational education, lower secondary vocational education, primary education, and no education).

### Statistical analyses

Mean and frequency differences between boys’ and girls’ characteristics were examined using independent-samples t tests for continuous variables and Chi-square statistics for categorical variables. Children were categorized into subgroups according to which measures identified them as overweight (‘overweight BMI and WC’, ‘overweight BMI and WHtR’, ‘overweight BMI only’, ‘overweight WC only’, ‘overweight WHtR only’) or which measures identified them as not overweight (‘not overweight BMI and WC’ and ‘not overweight BMI and WHtR’).

Mean and frequency differences were examined using analyses of variance (ANOVA) and Chi-square statistics to explore potential differences between the subgroups with regards to 1) children’s characteristics and 2) the healthcare professional’s clinical judgment on the weight status of the child. For all analyses examining differences between subgroups, a p-value < 0.05 was considered to be statistically significant. Agreement between BMI and WC and between BMI and the WHtR on the prevalence of overweight (obesity included) was investigated by cross-tabulation and expressed as observed proportion of agreement and Cohen’s kappa [27,28]. We regarded neither BMI, nor WC or WHtR as the ‘gold standard’. As we observed the paradox of a low kappa value and relatively high observed proportion of agreement, which is the result of the imbalance in the distribution of the marginal totals in the cross-table, we calculated a positive agreement index and a negative agreement index in addition to Cohen’s kappa (see Additional file 1 for calculations) [29,30]. Statistical analyses were performed using the SPSS program (version 15; SPSS Inc, Chicago, USA).

## Results

Among boys (n = 3895), the prevalence of overweight according to BMI cut-off points [21] was 7.0% (n = 272) and according to WC cut-off points [22] was 7.1% (n = 277) (Table 1 and Table 2). Of all boys, 4.3% (n = 168) were overweight according to both BMI and WC cut-off points, 4.3% (n = 168) were overweight according to both BMI and WHtR, 2.7% (n = 104) were overweight according to BMI only, 2.8% (n = 109) were overweight according to WC only, and 5.8% (n = 224) were overweight according to WHtR only (Table 2 and Table 4). In the subgroup of boys classified as overweight according to both BMI and WC (n = 168), 88.1% (n = 148) were also classified as overweight according to the clinical judgment of the healthcare professional. In the subgroup overweight BMI only (n = 104) and in the subgroup WC only (n = 109), respectively 66.3% (n = 69) and 16.5% (n = 18) of the children were also classified as overweight according to the clinical judgment of the healthcare professional (Table 1). In the subgroup classified as overweight according to both BMI and the WHtR (n = 168), 85.7% (n = 144) were also classified as overweight according to the clinical judgment of the healthcare professional. In the subgroup overweight BMI only (n = 104) and in the subgroup WHtR only (n = 224), respectively 70.2% (n = 73) and 7.6% (n = 17) of the children were also classified as overweight according to the clinical judgment of the healthcare professional (Table 3). The observed proportion of agreement between BMI and WC was 0.95, kappa was 0.58 (95% CI; 0.53-0.63), the observed proportion of positive agreement was 0.61, and the proportion of negative agreement was 0.97. The observed proportion of agreement between BMI and the WHtR was 0.92; kappa was 0.46 (95% CI; 0.41-0.51), the observed proportion of positive agreement was 0.51, and the proportion of negative agreement was 0.95.

**Table 1:** Characteristics of the study population and subgroups classified as overweight (obesity included) according to BMI, WC or both (n = 7703)

	**Boys**					
	**Total (n = 3895)**	**Overweight BMI and WC (n = 168)**	**Overweight BMI only (n = 104)**	**Overweight WC only (n = 109)**	**Not-overweight BMI and WC (n = 3514)**	***p*-value**
Mean age, years (SD)	5.8 (0.4)	5.8 (0.4)	5.7 (0.5)	5.7 (0.4)	5.8 (0.4)	0.026^a^
Mean height, cm (SD)	117.8 (5.5)	122.1 (5.6)	117.8 (5.9)	121.5 (5.2)	117.5 (5.4)	0.000^b^
Mean weight, kg (SD)	21.6 (3.1)	28.9 (3.9)	25.3 (2.8)	24.5 (2.4)	21.0 (2.5)	0.000^c^
Mean BMI, kg/(m)^2^ (SD)	15.5 (1.4)	19.3 (1.5)	18.1 (0.9)	16.5 (0.7)	15.2 (1.0)	0.000^d^
Mean WC, cm (SD)	53.4 (3.7)	63.3 (4.3)	55.9 (2.2)	59.8 (1.5)	52.6 (2.8)	0.000^d^
Non-Dutch ethnicity, n (%)	568 (14.6)	44 (26.2)	30 (28.8)	16 (14.7)	478 (13.6)	0.000^e^
Parental educational level low, n (%)	845 (21.7)	60 (35.7)	33 (31.7)	25 (22.9)	727 (20.7)	0.000^f^
Overweight according to clinical judgment of healthcare professional, n (%)	259 (6.6)	148 (88.1)	69 (66.3)	18 (16.5)	24 (0.7)	0.000^d^
	**Girls**					
	**Total (n = 3808)**	**Overweight BMI and WC (n = 258)**	**Overweight BMI only (n = 183)**	**Overweight WC only (n = 128)**	**Not-overweight BMI and WC (n = 3239)**	***p*-value**
Mean age, years (SD)	5.7 (0.4)^*^	5.8 (0.4)	5.7 (0.4)	5.8 (0.4)	5.7 (0.4)	0.751
Mean height, cm (SD)	117.0 (5.6)^**^	120.8 (5.7)	117.7 (5.5)	120.5 (5.2)	116.5 (5.5)	0.000^g^
Mean weight, kg (SD)	21.3 (3.4)^**^	27.9 (3.6)	24.9 (2.5)	23.8 (2.5)	20.5 (2.5)	0.000^h^
Mean BMI, kg/(m)^2^ (SD)	15.5 (1.6)	19.0 (1.5)	17.9 (0.8)	16.3 (0.9)	15.0 (1.1)	0.000^d^
Mean WC, cm (SD)	53.2 (4.1)^*^	62.4 (4.0)	55.7 (1.8)	59.9 (1.8)	52.1 (2.8)	0.000^d^
Non-Dutch ethnicity, n (%)	586 (15.4)	57 (22.1)	33 (18.0)	24 (18.8)	472 (14.6)	0.005^i^
Parental educational level low, n (%)	842 (22.1)	91 (35.3)	54 (29.5)	30 (23.4)	667 (20.6)	0.000^j^
Overweight according to clinical judgment of healthcare professional, n (%)	387 (10.2)^**^	233 (90.3)	113 (61.7)	16 (12.5)	25 (0.8)	0.000^d^

BMI = body mass index; WC = waist circumference.

^*^p < 0.05 for difference between boys and girls.

^**^p < 0.001 for difference between boys and girls.

Post-Hoc Tests (Tukey HSD for continuous determinants and cross-tabulation (2x2) for dichotomous determinants):

A = difference between ‘overweight BMI and WC’ and ‘BMI only’.

B = difference between ‘overweight BMI and WC’ and ‘WC only’.

C = difference between ‘overweight BMI and WC’ and ‘not-overweight BMI and WC’.

D = difference between ‘BMI only’ and ‘WC only’.

E = difference between ‘BMI only’ and ‘not-overweight BMI and WC’.

F = difference between ‘WC only’ and ‘not-overweight BMI and WC’.

^a^*p* < 0.05 for A and C.

^b^*p* < 0.001 for A, C, D and F.

^c^*p* < 0.001 for A, B, C, E, and F.

^d^*p* < 0.001 for A, B, C, D, E and F.

^e^*p* < 0.05 for B and D, *p* < 0.001 for C and E.

^f^*p* < 0.05 for B, D and F, *p* < 0.01 for E, *p* < 0.001 for C .

^g^*p* < 0.05 for E, *p* < 0.001 for A, C, D, and F.

^h^*p* < 0.01 for D, *p* < 0.001 for A, B, C, E and F.

^i^*p* < 0.01 for C.

^j^*p* < 0.05 for E, *p* < 0.001 for C.

**Table 2:** Comparison of weight status (not overweight/overweight, obesity included) according to BMI and WC cut-off points (n = 7703)

		**Boys (n = 3895)**			**Girls (n = 3808)**		
		**BMI**			**BMI**		
		**Overweight**	**Not overweight**	**Total**	**Overweight**	**Not overweight**	**Total**
**WC**	**Overweight**	168 (4.3%)	109 (2.8%)	277 (7.1%)	258 (6.8%)	128 (3.4%)	386 (10.1%)
	**Not overweight**	104 (2.7%)	3514 (90.2%)	3618 (92.9%)	183 (4.8%)	3239 (85.1%)	3422 (89.9)
	**Total**	272 (7.0%)	3623 (93.0%)	3895 (100%)	441 (11.6%)	3367 (88.4%)	3808 (100%)

BMI = body mass index; WC = waist circumference.

Percentages presented are total percentages.

Observed agreement: boys = 0.95; girls = 0.92.

Cohen’s kappa: boys = 0.58 (95% CI; 0.53 – 0.63); girls = 0.58 (95% CI; 0.54 -0.62).

Positive agreement: boys = 0.61; girls = 0.62.

Negative agreement: boys = 0.97; girls = 0.95.

(See Additional file 1 for calculations).

**Table 3:** Characteristics of the subgroups classified as overweight (obesity included) according to BMI, WHtR or both (n = 7703)

	**Boys (n = 3895)**				
	**Overweight BMI and WHtR (n = 168)**	**Overweight BMI only (n = 104)**	**Overweight WHtR only (n = 224)**	**Not-overweight BMI and WHtR (n = 3399)**	***p*-value**
Mean age, years (SD)	5.8 (0.5)	5.8 (0.4)	5.5 (0.4)	5.8 (0.4)	0.000^a^
Mean height, cm (SD)	119.8 (6.3)	121.6 (5.6)	113.3 (4.8)	117.9 (5.3)	0.000^b^
Mean weight, kg (SD)	27.9 (4.4)	27.0 (2.9)	21.0 (2.4)	21.1 (2.6)	0.000^c^
Mean BMI, kg/(m)^2^ (SD)	19.3 (1.5)	18.2 (0.9)	16.3 (0.8)	15.2 (1.0)	0.000^d^
Mean WC, cm (SD)	62.6 (5.0)	57.1 (2.9)	56.7 (2.7)	52.6 (2.8)	0.000^e^
Non-Dutch ethnicity, n (%)	56 (33.3)	18 (17.3)	39 (17.4)	455 (13.4)	0.000^f^
Parental educational level low, n (%)	57 (33.9)	36 (34.6)	48 (21.4)	704 (20.7)	0.000^c^
Overweight according to clinical judgment of healthcare professional, n (%)	144 (85.7)	73 (70.2)	17 (7.6)	25 (0.7)	0.000^g^
	**Girls (n = 3808)**				
	**Overweight BMI and WHtR (n = 216)**	**Overweight BMI only (n = 225)**	**Overweight WHtR only (n = 169)**	**Not-overweight BMI and WHtR (n = 3198)**	***p*-value**
Mean age, years (SD)	5.7 (0.4)	5.8 (0.4)	5.6 (0.4)	5.7 (0.4)	0.001^h^
Mean height, cm (SD)	118.2 (5.6)	120.8 (5.7)	112.9 (5.6)	116.9 (5.4)	0.000^i^
Mean weight, kg (SD)	26.8 (3.9)	26.5 (3.2)	20.7 (2.7)	20.6 (2.6)	0.000^c^
Mean BMI, kg/(m)^2^ (SD)	19.1 (1.6)	18.1 (0.8)	16.1 (0.9)	15.0 (1.1)	0.000^d^
Mean WC, cm (SD)	62.4 (4.6)	57.0 (2.7)	57.3 (3.1)	52.1 (2.9)	0.000^e^
Non-Dutch ethnicity, n (%)	50 (23.1)	40 (17.8)	31 (18.3)	465 (14.5)	0.003^j^
Parental educational level low, n (%)	74 (34.3)	71 (31.6)	40 (23.7)	657 (20.5)	0.000^k^
Overweight according to clinical judgment of healthcare professional, n (%)	197 (91.2)	149 (66.2)	14 (8.3)	27 (0.8)	0.000^d^

BMI = body mass index; WC = waist circumference; WHtR = waist-height ratio.

Post-Hoc Tests (Tukey HSD for continuous determinants and cross-tabulation (2x2) for dichotomous determinants):

A = difference between ‘overweight BMI and WHtR’ and ‘BMI only’.

B = difference between ‘overweight BMI and WHtR’ and ‘WHtR only’.

C = difference between ‘overweight BMI and WHtR’ and ‘not-overweight BMI and WHtR’.

D = difference between ‘BMI only’ and ‘WHtR only’.

E = difference between ‘BMI only’ and ‘not-overweight BMI and WHtR’.

F = difference between ‘WHtR only’ and ‘not-overweight BMI and WHtR’.

^a^*p* < 0.001 for B, D, and F.

^b^*p* < 0.05 for A, *p* < 0.001 for B, C, D, E, and F.

^c^*p* < 0.001 for B, C, D, and E.

^d^*p* < 0.001 for A, B, C, D, E and F.

^e^*p* < 0.001 for A, B, C, E and F.

^f^*p* < 0.05 for A, *p* < 0.01 for C, *p* < 0.001 for B.

^g^*p* < 0.01 for A, *p* < 0.001 for B, C, D, E, and F.

^h^*p* < 0.01 for F, *p* < 0.001 for D.

^i^*p* < 0.01 for C, *p* < 0.001 for A, B, D, E, and F.

^j^*p* < 0.001 for C.

^k^*p* < 0.05 for B, *p* < 0.01 for E, *p* < 0.001 for C.

**Table 4:** Comparison of weight status (not overweight/overweight, obesity included) according to BMI and WHtR cut-off points (n = 7703)

		**Boys (n = 3895)**			**Girls (n = 3808)**		
		**BMI**			**BMI**		
		**Overweight**	**Not overweight**	**Total**	**Overweight**	**Not overweight**	**Total**
**WHtR**	**Overweight**	168 (4.3%)	224 (5.8%)	392 (10.1%)	216 (5.7%)	169 (4.4%)	385 (10.1%)
	**Not overweight**	104 (2.7%)	3399 (87.3%)	3503 (89.9%)	225 (5.9%)	3198 (84.0%)	3423 (89.9%)
	**Total**	272 (7.0%)	3623 (93.0%)	3895 (100%)	441 (11.6%)	3367 (88.4%)	3808 (100%)

BMI = body mass index; WHtR = waist-height ratio.

Percentages presented are total percentages.

Observed agreement: boys = 0.92; girls = 0.90.

Cohen’s kappa: boys = 0.46 (95% CI; 0.41 – 0.51); girls = 0.47 (95% CI; 0.42 -0.51).

Positive agreement: boys = 0.51; girls = 0.52.

Negative agreement: boys = 0.95; girls = 0.94.

(See Additional file 1 for calculations).

Overall, results were similar for girls, but the prevalence of overweight was higher for girls compared to boys (according to BMI cut-off points the prevalence was 11.6% (n = 441) and according to WC cut-off points the prevalence was 10.1 % (n = 386)) (Table 1 and Table 2).

The subgroups of children classified as overweight according to both BMI and WC, BMI only or WC only differed in mean height, weight, BMI, WC, ethnic background, and parental educational level (Table 1). Compared to the other subgroups, children classified as overweight according to both BMI and WC were the tallest and heaviest and had the highest BMI and the largest WC. Further, children classified as overweight according to WC only were on average 3.0 cm taller than children classified as overweight according to BMI only (Table 1). The subgroups of children classified as overweight according to both BMI and WHtR, to BMI only or to WHtR only, also differed in mean height, weight, BMI, WC, ethnic background, and parental educational level (Table 3). These subgroups also differed in age; children classified as overweight according to WHtR only were relatively younger. Further, children classified as overweight according to WHtR only were relatively lighter in weight and shorter in height compared to other subgroups (Table 3).

## Discussion

We compared the assessment of overweight among 5-year-old children using BMI versus WC and BMI versus the WHtR. The results of the agreement analyses show that overall agreement between BMI classification versus WC and BMI classification versus the WHtR was high but the positive agreement between the measures was moderate to substantial. This indicated that BMI versus WC and BMI versus the WHtR agree moderately to substantially about the presence of overweight among 5-year-old children. It appeared that more than one third of the total group of children classified as overweight according to BMI was not classified as overweight according to WC or the WHtR. This was the same in the group of children classified as overweight according to WC. In the group of children classified as overweight according to the WHtR, more than half of the total group was classified as overweight only according to the WHtR.

Compared to the overweight BMI only group, children classified as overweight according to WC only were relatively taller and more boys were of Dutch ethnic background. When comparing BMI to WHtR, children classified as overweight according to WHtR only were relatively younger, shorter, and lighter. Approximately 2 in 3 of the children classified as overweight according to BMI only were also clinically judged as overweight by a healthcare professional during a well-child visit. In the subgroup classified as overweight according to WC only, this ratio was approximately 1 in 7 and in the subgroup classified as overweight according to WHtR only, this ratio was approximately 1 in 12.

Our data comes from a large population-based study of young children. Because of the small age range, our results are specific to the 5-year-old age group. The weight status of the children according to BMI was assessed using the IOTF’s age-specific and sex-specific cut-off points [21]. These cut-offs were chosen because they are used by the healthcare professionals at the municipal health services in the Netherlands [19]. By using these cut-off points, international comparisons of the prevalence of childhood overweight are also possible. Cut-off points for WC are not used in general by the healthcare professionals to assess children’s weight status during well-child visits. Also, no international validated cut-off points for WC are available. We used the available age-specific and sex-specific cut-off points for WC for Dutch children as presented by Fredriks et al. [22]. These cut-off points were based on data of 14,500 children aged 0–21 years in the Fourth Dutch Growth Study [22]. Internationally accepted cut-off points are also not available for the WHtR, and we used the 90^th^ percentile within our total study population at baseline (n = 8784) as the cut-off point for classification of overweight (obesity included). We also investigated the agreement between BMI and WHtR classification by using the cut-off point of 0.5 for the WHtR [16,17] instead of the 90^th^ percentile, and results were similar.

As reported in previous studies, more girls were classified as overweight compared to boys in our study [2,13,31,32]. As indicated in literature, a contribution to this may have been made by a significant difference in the sensitivity of the IOTF BMI cut-off points [2,4,13]. Children of parents with a low educational level, as an indicator of low socio-economic status [33], and children of non-Dutch ethnic background [34] are at increased risk for overweight and this is also reflected in our results. A relatively large number of the children in our study population were of non-Dutch ethnic background (main ethnicities: Moroccan, Turkish, Surinamese and Dutch Antillean), which allowed us to investigate differences in the distribution of ethnic background across the subgroups classified as overweight. The number of non-Dutch children was higher in the overweight subgroups but only among boys there was a statistically significant difference in ethnic background between the subgroups classified as overweight according to BMI only and WC only.

We compared the characteristics of the children in the study population with the characteristics of the children who were excluded from analyses due to missing data. Among boys, we found no statistically significant differences between those groups in age, height, weight, WC, ethnic background, parental educational level, or weight status of the children. Among girls, we found that weight, WC, and the prevalence of overweight according to BMI was higher among girls in the study population compared to girls who were excluded from analyses. However, we assume that the results of our analyses would be similar in the subgroup of girls with missing data.

Previous studies indicated that measuring only BMI results in an underestimation of health risk. To our knowledge, our study is the first study comparing classification results between BMI and WC and between BMI and the WHtR among 5-year-old children. The study by Fredriks et al. [22] found a strong correlation between BMI and WC. In additional analyses we also investigated the overall correlation in our study population, and we found comparable results. However, when we divided the BMI-group into quartiles, it appeared that the correlation was high only among children with a BMI in the highest quartile (see Additional file 2). So these findings indicate that BMI and WC merely agree among children with excess body fat in the highest percentile groups. This is also reflected in the results of the analyses in which we compared the children’s characteristics between the overweight subgroups; the children classified as overweight according to both BMI and WC had the highest amount of overall body fat (as estimated by BMI) and abdominal fat (as estimated by WC).

Further, we found that children classified as overweight according to both BMI and WC, and according to WC only, appeared to be relatively taller than the group classified as overweight according to BMI only. On the other hand, when comparing BMI and the WHtR, we found that children classified as overweight according to BMI only were relatively tall, and children classified as overweight according to WHtR only were short. There could be several explanations for these findings. First, it has been suggested that BMI is a measure of excess weight relative to height and not of excess body fat [14,35]. Therefore, BMI might not be a sensitive measure of body fat among children who are particularly short or tall or who have an unusual body fat distribution [36]. Second, children classified as overweight according to WC only might have a high WC as a consequence of being relatively tall for their age; this subgroup might include ‘overall large children’. We recommend future studies to investigate whether the WHtR can be used to assess overweight among relatively short children. Specifically, future studies should assess which cut-off points for WC or the WHtR are best to classify overweight among young children.

## Conclusions

The results of our study show that, overall, the adiposity markers BMI versus WC and BMI versus the WHtR are only in moderate agreement on the presence of overweight among 5-year-olds. They agree on overweight status among these young children in the higher percentiles of the overweight group. However, when prevention of further increase of excess body fat is considered, children with levels of BMI and WC near the norm are particularly important. This group consisted of children with overweight according to BMI only, WC only, and WHtR only. If BMI cut-off points continue to be used by healthcare professionals as a basis for their assessment of overweight among young children in monitoring programs, then part of the children classified as overweight according to WC will be omitted. Even though this is a small percentage of the total population, it is a relatively large percentage of the group of children identified as overweight according to WC or WHtR. This group of children might also be at increased risk for overweight-related health problems [8-11]. Our results show that BMI might not be a sensitive marker among relatively tall or short children and that WC should be measured in addition to BMI among these children.

We recommend future studies to compare the subgroups of children identified as overweight according to BMI only, WC only, and WHtR only over time and to examine these children’s risk of developing overweight-related health problems. It can then be decided whether WC should also be measured across the board in monitoring programs or only measured among certain subgroups such as relatively tall or short children. This will improve the identification and prevention of overweight and overweight-related health problems in children.

## Competing interests

The authors declare that they have no competing interests.

## Authors’ contributions

All authors have participated in the concept and design; analysis and interpretation of the data; drafting or revising of the manuscript: HR originated the idea for the study and its design and was responsible for acquiring the grant for the study. LV further developed the described the study protocol and was responsible for the data collection, data analyses and reporting of the results of the study. IV helped develop questionnaires, contributed to the data analyses and reporting of the results. WJ contributed to the analyses and the reporting of the results. CR and RH contributed to the design of the study, data collection procedures, plan for analysis, and reporting the results. HR supervised the study. All authors contributed to revision of the manuscript. All authors hereby confirm that the manuscript is an original piece of research. All authors have seen and approved the final version for publication and take full responsibility for the manuscript.

## Pre-publication history

The pre-publication history for this paper can be accessed here:

http://www.biomedcentral.com/1471-2431/13/63/prepub

## Supplementary Material

Additional file 1: Appendix 1 Calculation of kappa and proportions of agreement [27-30]. Click here for file

Additional file 2: Appendix 2 Mean levels of WC and correlation between BMI and WC among subgroups of BMI (n = 7703). Click here for file
