# Supplementary material for: Moderate agreement between body mass index and measures of waist circumference in the identification of overweight among 5-year-old children; the ‘Be active, eat right’ study
Source: BMC Pediatr. 2013 Apr 23;13:63. doi: 10.1186/1471-2431-13-63 (PMC3679730; doi:10.1186/1471-2431-13-63)
Supplement: Additional file 1: Appendix 1 — Calculation of kappa and proportions of agreement [27-30]. [file 1471-2431-13-63-S1.doc]

# Appendix 2 Mean levels of WC and correlation between BMI and WC among subgroups of BMI (n=7703)

|  | Boys (n=3895) | | | | | | | | | |
| --- | --- | --- | --- | --- | --- | --- | --- | --- | --- | --- |
|  | BMI | | | | | | | | | |
|  | Overall |  | Overweight (obesity included) | |  | | Percentiles | | | |
|  |  |  | No | Yes |  | < 25^th^ | | 25th – 49th | 50th – 74th | ≥ 75^th^ |
| WC |  |  |  |  |  |  | |  |  |  |
| Mean (SD) | 53.4 (3.7) |  | 52.8 (3.0) | 60.5 (5.1) |  | 50.7 (2.5) | | 52.4 (2.4) | 53.7 (2.5) | 56.8 (4.2) |
| Correlation | 0.73 |  | 0.58 | 0.67 |  | 0.27 | | 0.17 | 0.18 | 0.72 |
|  | Girls (n=3808) | | | | | | | | | |
|  | BMI | | | | | | | | | |
|  | Overall |  | Overweight (obesity included) | |  | | Percentiles | | | |
|  |  |  | No | Yes |  | < 25^th^ | | 25th – 49th | 50th – 74th | ≥ 75^th^ |
| WC |  |  |  |  |  |  | |  |  |  |
| Mean (SD) | 53.2 (4.1) |  | 52.4 (3.2) | 59.6 (4.6) |  | 50.2 (2.6) | | 51.9 (2.5) | 53.4 (2.5) | 57.4 (4.3) |
| Correlation | 0.76 |  | 0.60 | 0.71 |  | 0.28 | | 0.18 | 0.21 | 0.71 |

^a^ Differences in mean WC between groups overweight/non-overweight, and between different percentile-groups of BMI: all *p* < 0.001.

^b^ Pearson’s correlation coefficient *r*: all *p* < 0.01.

# 
